# Does it make a difference to add automatic EPAP titration to the volume-targeted pressure support mode in noninvasive ventilation of hypercapnic ICU patients?

**DOI:** 10.1186/cc14331

**Published:** 2015-03-16

**Authors:** G Gursel, A Zerman, B Basarik, K Gonderen, M Aydogdu, S Memmedova

**Affiliations:** 1 Gazi University Medical Faculty, Ankara, Turkey

## Introduction

Obese patients are increasing in number in ICUs and more than 90% of them also have sleep apnea syndrome. Variability in upper airway resistance during sleep and awakening periods makes it difficult to set EPAP in these patients. A new mode that automatically titrates EPAP according to upper airway resistance and IPAP according to target tidal volume may be more effective. The aim of this study is to evaluate whether adding automatic EPAP titration to the volume-targeted pressure support mode will provide any therapeutic advantages in hypercapnic ICU patients.

## Methods

The hypercapnic patients treated with average volume-assured pressure support-automatic EPAP (AVAPS-AE) mode (Group1 (G1)) were compared with those treated with AVAPS mode (Group 2 (G2)). G2 was recruited retrospectively and matched with G1 according to diagnoses, demographic characteristics, arterial blood gas values and daily noninvasive ventilation (NIV) usage times. Trilogy 100® devices and their software Directview® (Philips Respironics) were used to reveal the respiratory data such as pressures, volumes, and daily usage times. For statistical analyses, *t *test, chi-square test and repeated measures of ANOVA were used.

## Results

Twenty-eight patients were included in G1 and 22 patients in G2. There was no significant difference between the patients' admission parameters and daily NIV usage times. PaCO_2_ decreased >5 mmHg in 93% of G1 patients and in 60% of G2 patients in the first 6 hours (*P *= 0.044). A 10 mmHg reduction in PaCO_2_ occurred in more patients (93% vs. 60%, *P *= 0.004) and in a shorter time (1.8 ± 1.2 vs. 3 ± 3 days, *P *= 0.044) in G1. At the time of discharge, PaCO_2_ levels were <50 mmHg in 79% of G1 and 41% of G2 patients (*P *= 0.006). Both groups showed similar and significant improvements in PaO_2_, PaCO_2_ and HCO_3_^-^ levels within the first 4 days but only in G1 patients were HCO_3_^-^ levels decreased more rapidly than G2 patients (*P = *0.007). Duration of NIV (6 ± 2 vs. 8 ± 3 days, *P = *0.002) and the number of mode and pressures changes (0.3 ± 1.8 vs. 2 ± 2 times, *P >*0.0001) were significantly less in G1. While mean IPAP was similar in both groups, maximum and minimum EPAP titrated automatically in G1 were significantly different from G2. Mean tidal volume and amount of leakage were also significantly higher in G1.

## Conclusion

These results suggest that the AVAPS-AE mode may provide some advantages in hypercapnic ICU patients such as rapid PaCO_2_ reduction, less NIV duration and workload.

